# Association of maternal sleep before and during pregnancy with sleep and developmental problems in 1-year-old infants

**DOI:** 10.1038/s41598-021-91271-7

**Published:** 2021-06-04

**Authors:** Kazushige Nakahara, Takehiro Michikawa, Seiichi Morokuma, Masanobu Ogawa, Kiyoko Kato, Masafumi Sanefuji, Eiji Shibata, Mayumi Tsuji, Masayuki Shimono, Toshihiro Kawamoto, Shouichi Ohga, Koichi Kusuhara, the Japan Environment and Children’s Study Group, Michihiro Kamijima, Shin Yamazaki, Yukihiro Ohya, Reiko Kishi, Nobuo Yaegashi, Koichi Hashimoto, Chisato Mori, Shuichi Ito, Zentaro Yamagata, Hidekuni Inadera, Takeo Nakayama, Hiroyasu Iso, Masayuki Shima, Youichi Kurozawa, Narufumi Suganuma, Takahiko Katoh

**Affiliations:** 1 Department of Obstetrics and Gynecology, Graduate School of Medical Sciences, Kyushu University, Fukuoka, Japan; 2 Department of Environmental and Occupational Health, School of Medicine, Toho University, Tokyo, Japan; 3 Department of Health Sciences, Graduate School of Medical Sciences, Kyushu University, Fukuoka, 812-8582 Japan; 4 Research Center for Environmental and Developmental Medical Sciences, Kyushu University, Fukuoka, Japan; 5 Department of Pediatrics, Graduate School of Medical Sciences, Kyushu University, Fukuoka, Japan; 6 Regional Center for Japan Environment and Children’s Study, University of Occupational and Environmental Health, Kitakyushu, Japan; 7 Department of Obstetrics and Gynecology, School of Medicine, University of Occupational and Environmental Health, Kitakyushu, Fukuoka Japan; 8 Department of Environmental Health, School of Medicine, University of Occupational and Environmental Health, Kitakyushu, Fukuoka Japan; 9 Department of Pediatrics, School of Medicine, University of Occupational and Environmental Health, Kitakyushu, Fukuoka Japan; 10 Department of Occupational and Environmental Health, Graduate School of Medical Sciences, Nagoya City University, 1 Kawasumi, Mizuho-cho, Mizuho-ku, Nagoya, Aichi 467-8601 Japan; 11 National Institute for Environmental Studies, Tsukuba, Japan; 12 National Center for Child Health and Development, Tokyo, Japan; 13 Hokkaido University, Sapporo, Japan; 14 Tohoku University, Sendai, Japan; 15 Fukushima Medical University, Fukushima, Japan; 16 Chiba University, Chiba, Japan; 17 Yokohama City University, Yokohama, Japan; 18 University of Yamanashi, Chuo, Japan; 19 University of Toyama, Toyama, Japan; 20 Kyoto University, Kyoto, Japan; 21 Osaka University, Suita, Japan; 22 Hyogo College of Medicine, Nishinomiya, Japan; 23 Tottori University, Yonago, Japan; 24 Kochi University, Nankoku, Japan; 25 Kumamoto University, Kumamoto, Japan

**Keywords:** Medical research, Risk factors

## Abstract

This study investigated the association of maternal sleep before and during pregnancy with sleeping and developmental problems in 1-year-old infants. We used data from the Japan Environment and Children’s Study, which registered 103,062 pregnancies between 2011 and 2014. Participants were asked about their sleep habits prior to and during pregnancy. Follow-up assessments were conducted to evaluate the sleep habits and developmental progress of their children at the age of 1 year. Development during infancy was evaluated using the Ages and Stages Questionnaire (ASQ). Maternal short sleep and late bedtime before and during pregnancy increased occurrence of offspring’s sleeping disturbances. For example, infants whose mothers slept for less than 6 h prior to pregnancy tended to be awake for more than 1 h (risk ratio [RR] = 1.49, 95% confidence interval [CI] 1.34–1.66), sleep less than 8 h during the night (RR = 1.60, 95% CI 1.44–1.79), and fall asleep at 22:00 or later (RR = 1.33, 95% CI 1.26–1.40). Only subjective assessments of maternal sleep quality during pregnancy, such as very deep sleep and feeling very good when waking up, were inversely associated with abnormal ASQ scores in 1-year-old infants.

## Introduction

Sleep duration among the general population in Japan has been reported to be shorter than that in other countries^1^ and has become even shorter in recent years^2^. Furthermore, it has been reported that approximately 10% of infants have sleeping problems^3^. Neurodevelopmental disorders, including autism spectrum disorder (ASD), neurodevelopment abnormalities, and disturbed sleep habits, such as late bedtime and intense night crying, are observed in early infancy^4^. The incidence of developmental disorders is increasing in developed countries, including Japan^5–7^. Factors related to developmental disorders include genetic ones and environmental (in utero) ones^8,9^. Maternal lifestyle such as sleep pattern may affect the offspring’s sleep and development.

It has been reported that maternal sleep disorders are associated with developmental progress in the offspring. For example, maternal sleep disordered breathing (SDB) during pregnancy is associated with the offspring’s development, manifesting as disrupted social skills and low reading-test scores^10,11^. Thus, not only maternal sleep habit but also maternal sleep disorders during pregnancy may be related to early infant sleep patterns and development. However, no large-scale study has examined the potential associations between maternal sleep and the offspring’s sleep patterns or development. Additionally, the importance of maternal sleep during various periods of pregnancy and the persistence of the influence remain unclear.

We previously reported that maternal sleep habits, such as short sleep duration and late bedtime, both before and during pregnancy, were associated with the offspring’s sleep problems and temperament at 1 month of age^12^. We hypothesize that maternal sleep before and during pregnancy would continue to be associated with the infant’s sleep and developmental problems even at 1 year of age.

This study aimed to expand on those findings and investigate the association between maternal sleep habits, before and during pregnancy, with offspring outcomes at 1 year of age.

## Results

The baseline characteristics of the participants, along with the available data on sleep duration before pregnancy, are shown in Table 1. The characteristics of participants in the various sleep groups are also shown in Supplemental Table 1. The reported sleep duration was on average between 7 and 8 h, both before and during pregnancy. The participants tended to sleep longer and go to bed earlier during pregnancy than before pregnancy. Significant data points are summarized below and include risk ratios (RR) and 95% confidence intervals (CI) in the multivariable model, adjusted for maternal age at delivery, smoking habits, alcohol consumption, pre-pregnancy body mass index, gestational age at birth, parity, infertility treatment, and infant sex.

**Table 1:** Baseline characteristics of the study population stratified by sleep duration before pregnancy.

	Maternal sleep before pregnancy (h)											
	< 6 h		6 < 7		7 < 8		8 < 9		9 < 10		10<	
	n	%	n	%	n	%	n	%	n	%	n	%
Maternal sleep	4948	6.7	14,661	19.9	25,136	34.1	18,302	24.8	7494	10.2	3286	4.5
**Maternal characteristics**												
Age at delivery (years)												
< 25	490	9.9	1025	7.0	1825	7.3	1539	8.4	827	11.0	786	23.9
25–29	1258	25.4	3940	26.9	6968	27.7	5087	27.8	2154	28.7	1006	30.6
30–34	1602	32.4	5278	36.0	9266	36.9	6806	37.2	2698	36.0	937	28.5
≥ 35	1598	32.3	4418	30.1	7077	28.2	4870	26.6	1815	24.2	557	17.0
Smoking habits												
Never smoked	2708	54.7	9026	61.6	15,537	61.8	10,935	59.8	4304	57.4	1606	48.9
Ex-smokers who quit before pregnancy	1049	21.2	3193	21.8	5787	23.0	4550	24.9	1967	26.3	818	24.9
Smokers during early pregnancy	1191	24.1	2442	16.7	3812	15.2	2817	15.4	1223	16.3	862	26.2
Alcohol consumption												
Never drank	1575	31.8	4819	32.9	8628	34.3	6552	35.8	2727	36.4	1193	36.3
Ex-drinkers who quit before pregnancy	799	16.2	2365	16.1	4321	17.2	3546	19.4	1615	21.6	680	20.7
Drinkers during early pregnancy	2574	52.0	7477	51.0	12187	48.5	8204	44.8	3152	42.1	1413	43.0
Pre-pregnancy body mass index (kg/m^2^)												
< 18.5	790	16.0	2349	16.0	4023	16.0	2949	16.1	1188	15.9	586	17.8
18.5–24.9	3618	73.1	10,909	74.4	18,658	74.2	13,549	74.0	5534	73.9	2367	72.0
≥ 25.0	540	10.9	1403	9.6	2455	9.8	1804	9.9	772	10.3	333	10.1
Parity												
0	2982	60.3	8643	59.0	12,176	48.4	5950	32.5	1748	23.3	1326	40.4
≥ 1	1966	39.7	6018	41.1	12,960	51.6	12,352	67.5	5746	76.7	1960	59.7
Infertility treatment												
No	4573	92.4	13,482	92.0	23,252	92.5	17,197	94.0	7175	95.7	3158	96.1
Ovulation stimulation/artificial insemination by sperm from husband	202	4.1	641	4.4	1005	4.0	640	3.5	190	2.5	72	2.2
Assisted reproductive technology	173	3.5	538	3.7	879	3.5	465	2.5	129	1.7	56	1.7
Gestational age (weeks)												
Early term (37–38)	1565	31.6	4573	31.2	8099	32.2	6164	33.7	2581	34.4	1070	32.6
Full term (39–41)	3383	68.4	10,088	68.8	17,037	67.8	12,138	66.3	4913	65.6	2216	67.4
Infant sex												
Boys	2508	50.7	7434	50.7	12,758	50.8	9323	50.9	3879	51.8	1686	51.3
Girls	2440	49.3	7227	49.3	12,378	49.2	8979	49.1	3615	48.2	1600	48.7

### Maternal sleep before pregnancy and infant sleep (Table 2)

**Table 2:** Association between sleep before pregnancy and infant sleep, Japan Environment and Children’s Study (2011–2014).

	No. of participants	No. of outcome		Maternal age adjusted model			Multivariable model^a^		
			%	RR	95% CI		RR	95% CI	
**> 3 nighttime waking instances**									
Sleep duration									
< 6 h	4948	118	2.4	1.04	0.85	1.26	1.08	0.89	1.32
6 < 7	14,661	324	2.2	0.95	0.83	1.09	0.96	0.84	1.10
7 < 8	25,136	584	2.3	Ref			Ref		
8 < 9	18,302	466	2.6	1.10	0.98	1.24	1.09	0.96	1.23
9 < 10	7494	209	2.8	1.23	1.05	1.43	1.20	1.02	1.40
10<	3286	74	2.3	1.06	0.84	1.35	1.07	0.84	1.36
Bedtime									
21 < 24	49,995	1275	2.6	Ref			Ref		
24 < 27	21,825	461	2.1	0.85	0.76	0.94	0.89	0.80	0.99
Other	2007	39	1.9	0.83	0.61	1.14	0.90	0.65	1.23
**> 1 waking instances lasting > 1 h**									
Sleep duration									
< 6 h	4948	411	8.3	1.54	1.38	1.71	1.49	1.34	1.66
6 < 7	14,661	929	6.3	1.18	1.09	1.28	1.16	1.07	1.26
7 < 8	25,136	1343	5.3	Ref			Ref		
8 < 9	18,302	916	5.0	0.94	0.86	1.02	0.96	0.89	1.04
9 < 10	7494	387	5.2	0.96	0.86	1.07	1.00	0.90	1.12
10<	3286	224	6.8	1.24	1.08	1.42	1.25	1.09	1.44
Bedtime									
21 < 24	49,995	2460	4.9	Ref			Ref		
24 < 27	21,825	1550	7.1	1.44	1.35	1.53	1.38	1.30	1.47
Other	2007	200	10.0	1.99	1.73	2.28	1.92	1.67	2.21
**< 8 h of sleep during the night (20:00–7:59)**									
Sleep duration									
< 6 h	4948	408	8.3	1.65	1.48	1.84	1.60	1.44	1.79
6 < 7	14,661	872	6.0	1.21	1.11	1.31	1.19	1.09	1.29
7 < 8	25,136	1233	4.9	Ref			Ref		
8 < 9	18,302	804	4.4	0.89	0.82	0.98	0.92	0.84	1.00
9 < 10	7494	319	4.3	0.86	0.77	0.97	0.90	0.80	1.02
10<	3286	207	6.3	1.25	1.08	1.44	1.26	1.09	1.46
Bedtime									
21 < 24	49,995	2284	4.6	Ref			Ref		
24 < 27	21,825	1361	6.2	1.37	1.28	1.46	1.31	1.22	1.40
Other	2007	198	9.9	2.11	1.84	2.43	2.04	1.77	2.35
**Falling asleep at 22:00 or later**									
Sleep duration									
< 6 h	4948	1363	27.6	1.39	1.32	1.46	1.33	1.26	1.40
6 < 7	14,661	3359	22.9	1.17	1.13	1.22	1.15	1.10	1.19
7 < 8	25,136	4898	19.5	Ref			Ref		
8 < 9	18,302	3348	18.3	0.94	0.90	0.97	0.96	0.92	1.00
9 < 10	7494	1183	15.8	0.80	0.76	0.85	0.84	0.79	0.89
10<	3286	691	21.0	1.02	0.95	1.10	1.02	0.95	1.10
Bedtime									
21 < 24	49,995	8412	16.8	Ref			Ref		
24 < 27	21,825	5935	27.2	1.60	1.55	1.65	1.53	1.48	1.58
Other	2007	495	24.7	1.40	1.29	1.52	1.34	1.23	1.45
**Frequency of crying at night (≥ 5 days/week)**									
Sleep duration									
< 6 h	4948	395	8.0	1.14	1.03	1.27	1.16	1.05	1.29
6 < 7	14,661	1085	7.4	1.05	0.98	1.13	1.05	0.98	1.13
7 < 8	25,136	1771	7.1	Ref			Ref		
8 < 9	18,302	1345	7.4	1.05	0.98	1.12	1.05	0.98	1.12
9 < 10	7494	548	7.3	1.04	0.95	1.15	1.05	0.96	1.15
10<	3286	203	6.2	0.91	0.79	1.04	0.93	0.80	1.07
Bedtime									
21 < 24	49,995	3591	7.2	Ref			Ref		
24 < 27	21,825	1609	7.4	1.03	0.98	1.09	1.05	0.99	1.11
Other	2007	147	7.3	1.06	0.90	1.24	1.11	0.95	1.31

*CI* confidence interval, *RR* risk ratio. **Bold fonts** showed the items of infant's sleep outcomes.

^a^Adjusted for maternal age at delivery, smoking habits, alcohol consumption, pre-pregnancy body mass index, gestational age at birth, parity, infertility treatment, and infant sex.

Short sleep duration less than 6 h before pregnancy was associated with a higher risk of night waking for ≥ 1 h (RR = 1.49, 95% CI 1.34–1.66), sleeping for < 8 h at night (RR = 1.60, 95% CI 1.44–1.79), and falling sleep at 22:00 or later (RR = 1.33, 95% CI 1.26–1.40) in the offspring, compared to offspring of mothers who slept for 7–8 h. On the contrary, compared to offspring of mothers who slept for 7–8 h, the offspring of mothers who slept for more than 10 h before pregnancy were also at a higher risk of night waking for > 1 h (RR = 1.25, 95% CI 1.09–1.44) and sleeping for < 8 h at night (RR = 1.26, 95% CI 1.09–1.46). Compared to offspring of mothers who slept before midnight before pregnancy, offspring of mothers who slept after midnight had a higher risk of night waking for > 1 h (RR = 1.38, 95% CI 1.30–1.47), sleeping for < 8 h at night (RR = 1.31, 95% CI 1.22–1.40), and falling asleep at 22:00 or later (RR = 1.53, 95% CI 1.48–1.58).

In the sub-analysis limited to participants who slept for 7–9 h during pregnancy, we found similar associations between maternal sleep before pregnancy and the offspring’s sleep outcome (Supplemental Table 2). Maternal sleep for < 6 h increased the risk ratio of infants wakening for more than 1 h, sleeping for < 8 h during the night, and falling asleep at 22:00 or after. Maternal sleep for more than 10 h also increased the risk ratio of infants awakening for > 1 h and sleeping for < 8 h during the night. Maternal bedtime after midnight increased the risk ratio of infants awakening > 1 h, sleeping < 8 h, and falling asleep at 22:00 or later.

### Maternal sleep during pregnancy and infant sleep (Table 3)

**Table 3:** Association between sleep during pregnancy and infant sleep, Japan Environment and Children's Study (2011–2014).

	No. of participants	No. of outcome		Maternal age adjusted model			Multivariable model^a^		
			%	RR	95% CI		RR	95% CI	
**> 3 nighttime waking instances**									
Sleep duration									
< 6 h	3540	80	2.3	0.97	0.77	1.22	1.00	0.79	1.26
6 < 7	11,099	244	2.2	0.93	0.80	1.08	0.94	0.81	1.09
7 < 8	23,050	547	2.4	Ref			Ref		
8 < 9	21,043	492	2.3	0.99	0.88	1.11	0.98	0.86	1.10
9 < 10	10,394	288	2.8	1.19	1.03	1.37	1.16	1.01	1.34
10<	4701	124	2.6	1.20	0.99	1.46	1.21	0.99	1.46
Bedtime									
21 < 24	54,403	1361	2.5	Ref			Ref		
24 < 27	17,798	376	2.1	0.86	0.77	0.97	0.90	0.80	1.01
Other	1626	38	2.3	0.98	0.71	1.35	1.02	0.74	1.40
Depth of sleep									
Very light	5054	172	3.4	1.73	1.46	2.04	1.74	1.47	2.06
Light	30,977	888	2.9	1.45	1.31	1.61	1.44	1.30	1.60
Normal	29,459	579	2.0	Ref			Ref		
Deep	7007	117	1.7	0.85	0.70	1.04	0.86	0.70	1.04
Very deep	1330	19	1.4	0.74	0.47	1.16	0.75	0.47	1.18
Feeling when waking up in the morning									
Very bad	1112	38	3.4	1.50	1.09	2.07	1.55	1.13	2.13
Bad	15,106	425	2.8	1.19	1.06	1.32	1.19	1.07	1.33
Normal	45,965	1102	2.4	Ref			Ref		
Good	10,314	184	1.8	0.74	0.63	0.86	0.73	0.63	0.85
Very good	1330	26	2.0	0.81	0.55	1.20	0.82	0.56	1.21
**> 1 waking instances lasting > 1 h**									
Sleep duration									
< 6 h	3540	305	8.6	1.57	1.39	1.77	1.53	1.35	1.72
6 < 7	11,099	697	6.3	1.15	1.05	1.26	1.13	1.03	1.24
7 < 8	23,050	1254	5.4	Ref			Ref		
8 < 9	21,043	1119	5.3	0.98	0.90	1.06	1.00	0.93	1.09
9 < 10	10,394	550	5.3	0.97	0.88	1.07	1.01	0.92	1.12
10<	4701	285	6.1	1.09	0.96	1.23	1.10	0.97	1.25
Bedtime									
21 < 24	54,403	2730	5.0	Ref			Ref		
24 < 27	17,798	1321	7.4	1.47	1.38	1.57	1.41	1.32	1.51
Other	1626	159	9.8	1.93	1.66	2.25	1.90	1.63	2.21
Depth of sleep									
Very light	5054	339	6.7	1.23	1.10	1.37	1.24	1.11	1.39
Light	30,977	1794	5.8	1.07	1.00	1.14	1.08	1.01	1.15
Normal	29,459	1604	5.4	Ref			Ref		
Deep	7007	402	5.7	1.05	0.95	1.17	1.04	0.94	1.16
Very deep	1330	71	5.3	0.97	0.77	1.23	0.96	0.76	1.21
Feeling when waking up in the morning									
Very bad	1112	84	7.6	1.32	1.07	1.63	1.30	1.06	1.61
Bad	15,106	936	6.2	1.10	1.02	1.18	1.10	1.02	1.18
Normal	45,965	2582	5.6	Ref			Ref		
Good	10,314	546	5.3	0.94	0.86	1.03	0.94	0.86	1.03
Very good	1330	62	4.7	0.83	0.65	1.06	0.82	0.64	1.05
**< 8 h of sleep during the night (20:00–7:59)**									
Sleep duration									
< 6 h	3540	314	8.9	1.70	1.51	1.91	1.66	1.48	1.88
6 < 7	11,099	714	6.4	1.25	1.14	1.36	1.23	1.12	1.34
7 < 8	23,050	1184	5.1	Ref			Ref		
8 < 9	21,043	906	4.3	0.84	0.77	0.91	0.86	0.79	0.93
9 < 10	10,394	480	4.6	0.90	0.81	1.00	0.93	0.84	1.03
10<	4701	245	5.2	1.00	0.87	1.14	1.01	0.88	1.16
Bedtime									
21 < 24	54,403	2508	4.6	Ref			Ref		
24 < 27	17,798	1205	6.8	1.47	1.38	1.57	1.41	1.32	1.51
Other	1626	130	8.0	1.72	1.45	2.03	1.69	1.43	2.01
Depth of sleep									
Very light	5054	349	6.9	1.36	1.22	1.52	1.38	1.23	1.55
Light	30,977	1640	5.3	1.05	0.98	1.13	1.07	1.00	1.14
Normal	29,459	1477	5.0	Ref			Ref		
Deep	7007	316	4.5	0.90	0.80	1.02	0.89	0.79	1.00
Very deep	1330	61	4.6	0.91	0.71	1.17	0.90	0.70	1.15
Feeling when waking up in the morning									
Very bad	1112	92	8.3	1.63	1.34	2.00	1.62	1.33	1.98
Bad	15,106	934	6.2	1.25	1.16	1.34	1.25	1.16	1.34
Normal	45,965	2284	5.0	Ref			Ref		
Good	10,314	477	4.6	0.93	0.85	1.03	0.93	0.84	1.02
Very good	1330	56	4.2	0.84	0.65	1.09	0.83	0.64	1.08
**Falling asleep at 22:00 or later**									
Sleep duration									
< 6 h	3540	1029	29.1	1.43	1.35	1.51	1.39	1.31	1.47
6 < 7	11,099	2682	24.2	1.20	1.15	1.25	1.18	1.13	1.23
7 < 8	23,050	4617	20.0	Ref			Ref		
8 < 9	21,043	3720	17.7	0.88	0.85	0.92	0.90	0.87	0.94
9 < 10	10,394	1740	16.7	0.83	0.79	0.87	0.86	0.82	0.91
10<	4701	1054	22.4	1.07	1.01	1.14	1.08	1.02	1.15
Bedtime									
21 < 24	54,403	9402	17.3	Ref			Ref		
24 < 27	17,798	5120	28.8	1.65	1.60	1.70	1.58	1.53	1.63
Other	1626	320	19.7	1.12	1.01	1.24	1.10	0.99	1.21
Depth of sleep									
Very light	5054	1087	21.5	1.07	1.01	1.14	1.08	1.02	1.15
Light	30,977	6213	20.1	1.01	0.98	1.04	1.02	0.99	1.05
Normal	29,459	5882	20.0	Ref			Ref		
Deep	7007	1404	20.0	1.00	0.95	1.06	0.99	0.94	1.04
Very deep	1330	256	19.3	0.95	0.85	1.06	0.93	0.84	1.04
Feeling when waking up in the morning									
Very bad	1112	268	24.1	1.16	1.04	1.29	1.14	1.03	1.27
Bad	15,106	3341	22.1	1.10	1.06	1.14	1.10	1.06	1.14
Normal	45,965	9215	20.1	Ref			Ref		
Good	10,314	1804	17.5	0.88	0.84	0.92	0.88	0.84	0.92
Very good	1330	214	16.1	0.80	0.71	0.91	0.80	0.70	0.90
**Frequency of crying at night (≥ 5 days/week)**									
Sleep duration									
< 6 h	3540	256	7.2	0.99	0.88	1.13	1.01	0.89	1.14
6 < 7	11,099	848	7.6	1.04	0.96	1.13	1.05	0.97	1.13
7 < 8	23,050	1695	7.4	Ref			Ref		
8 < 9	21,043	1480	7.0	0.96	0.89	1.02	0.96	0.90	1.03
9 < 10	10,394	761	7.3	1.00	0.92	1.09	1.00	0.92	1.09
10<	4701	307	6.5	0.91	0.81	1.02	0.92	0.82	1.04
Bedtime									
21 < 24	54,403	3881	7.1	Ref			Ref		
24 < 27	17,798	1351	7.6	1.07	1.01	1.14	1.08	1.02	1.15
Other	1626	115	7.1	1.01	0.85	1.21	1.04	0.87	1.24
Depth of sleep									
Very light	5054	482	9.5	1.50	1.36	1.65	1.52	1.38	1.67
Light	30,977	2470	8.0	1.25	1.18	1.32	1.25	1.18	1.32
Normal	29,459	1882	6.4	Ref			Ref		
Deep	7007	431	6.2	0.96	0.87	1.07	0.96	0.87	1.06
Very deep	1330	82	6.2	0.97	0.78	1.20	0.97	0.79	1.20
Feeling when waking up in the morning									
Very bad	1112	111	10.0	1.43	1.20	1.72	1.46	1.22	1.75
Bad	15,106	1294	8.6	1.21	1.13	1.28	1.21	1.14	1.29
Normal	45,965	3275	7.1	Ref			Ref		
Good	10,314	596	5.8	0.81	0.74	0.88	0.80	0.74	0.87
Very good	1330	71	5.3	0.75	0.60	0.94	0.75	0.60	0.95

*CI* confidence interval, *RR* risk ratio. **Bold fonts** showed the items of infant's sleep outcomes.

^a^Adjusted for maternal age at delivery, smoking habits, alcohol consumption, pre-pregnancy body mass index, gestational age at birth, parity, infertility treatment, and infant sex.

As for the analysis of maternal sleep before pregnancy, short or long sleep duration and sleeping after midnight during pregnancy were associated with a higher risk of some sleep outcomes. Infants whose mothers slept for less than 6 h during pregnancy tended to be awake for > 1 h at night (RR = 1.53, 95% CI 1.35–1.72), to sleep for < 8 h during night (RR = 1.66, 95% CI 1.48–1.88), and to sleep at 22:00 or later (RR = 1.39, 95% CI 1.31–1.47), compared to the infants whose mother slept for 7–8 h. On the contrary, compared to the offspring of mothers who slept for 7–8 h, offspring of mothers who slept for more than 10 h during pregnancy tended to sleep at 22:00 or later (RR = 1.08, 95% CI 1.02–1.15). Maternal bedtime after midnight during pregnancy was also associated with a higher risk of infants night waking for > 1 h (RR = 1.41, 95% CI 1.32–1.51), sleeping for < 8 h at night (RR = 1.41, 95% CI 1.32–1.51), falling asleep at 22:00 or later (RR = 1.58, 95% CI 1.53–1.63), and frequency of crying (RR = 1.08, 95% CI 1.02–1.15), compared to the group of maternal bedtime before midnight.

In the sub-analysis limited to the participants who slept for 7–9 h before pregnancy, we found similar associations between maternal sleep during pregnancy and infants’ sleep outcome (Supplemental Table 2). Maternal sleep for less than 6 h and for more than 10 h increased the risk ratio of falling asleep at 22:00 or after. Maternal bedtime after midnight increased the risk ratio of infants awakening for > 1 h at night, sleep < 8 h at night, and falling asleep at 22:00 or later.

Subjective items of sleep during pregnancy were also associated with the offspring’s sleeping problems. For example, maternal “very light” sleep was associated with a higher risk of 3 or more waking instances in a night (RR = 1.74, 95% CI 1.47–2.06), night waking for more than 1 h (RR = 1.24, 95% CI 1.11–1.39), sleeping for less than 8 h at night (RR = 1.38, 95% CI 1.23–1.55), sleeping at 22:00 or later (RR = 1.08, 95% CI 1.02–1.15), crying 5 days or more in a week (RR = 1.52, 95% CI 1.38–1.67), compared to the group of maternal “normal” sleep depth.

### Maternal sleep and offspring developmental progress

We used the Japanese version of the Ages and Stages Questionnaire, third edition (J-ASQ-3), to evaluate the offspring’s development. There were no associations between sleep duration or bedtime, both before and during pregnancy, and abnormal J-ASQ-3 scores (Table 4). However, “good” and “very good” feelings when waking up during pregnancy were associated with a lower risk of abnormal J-ASQ-3 scores for any one of the five domains in a multivariable model (RR for good *vs.* normal = 0.86, 95% CI 0.81–0.91; RR for very good feeling *vs.* normal = 0.81, 95% CI 0.69–0.95) (Table 5), compared to the group of maternal “normal” feelings at waking up. Moreover, for the depth of sleep during pregnancy, “very deep” sleep decreased the risk of abnormal J-ASQ-3 scores (RR for very deep *vs.* normal = 0.83, 95% CI 0.71–0.98), compared to the group of maternal “normal” sleep depth.

**Table 4:** Association between sleep before pregnancy and infant development, Japan Environment and Children's Study (2011–2014).

	No. of participants	No. of outcome		Maternal age adjusted model			Multivariable model^a^		
			%	RR	95% CI		RR	95% CI	
**Communication**									
Sleep duration									
< 6 h	4489	5	0.1	**1.19**	0.45	3.15	**1.15**	0.43	3.03
6 < 7	13,427	18	0.1	**1.40**	0.75	2.61	**1.32**	0.71	2.47
7 < 8	23,058	22	0.1	**Ref**			**Ref**		
8 < 9	16,779	13	0.1	**0.82**	0.41	1.63	**0.90**	0.45	1.80
9 < 10	6863	6	0.1	**0.95**	0.39	2.35	**1.12**	0.45	2.82
10<	2975	3	0.1	**1.29**	0.38	4.31	**1.42**	0.42	4.78
Bedtime									
21 < 24	45,971	46	0.1	**Ref**			**Ref**		
24 < 27	19,816	19	0.1	**1.01**	0.59	1.73	**0.86**	0.49	1.50
Other	1804	2	0.1	**1.33**	0.32	5.51	**1.39**	0.33	5.79
**Gross motor skills**									
Sleep duration									
< 6 h	4489	265	5.9	**1.08**	0.95	1.23	**1.07**	0.94	1.22
6 < 7	13,427	832	6.2	**1.12**	1.03	1.22	**1.11**	1.02	1.20
7 < 8	23,066	1264	5.5	**Ref**			**Ref**		
8 < 9	16,775	902	5.4	**0.99**	0.91	1.07	**1.02**	0.93	1.10
9 < 10	6861	327	4.8	**0.89**	0.79	1.01	**0.94**	0.84	1.06
10<	2975	120	4.0	**0.82**	0.69	0.99	**0.86**	0.72	1.03
Bedtime									
21 < 24	45,972	2546	5.5	**Ref**			**Ref**		
24 < 27	19,816	1073	5.4	**1.01**	0.95	1.09	**0.97**	0.90	1.05
Other	1805	91	5.0	**1.01**	0.83	1.24	**1.06**	0.86	1.30
**Fine motor skills**									
Sleep duration									
< 6 h	4489	252	5.6	**1.01**	0.88	1.15	**1.02**	0.90	1.17
6 < 7	13,415	781	5.8	**1.04**	0.95	1.13	**1.05**	0.97	1.15
7 < 8	23,063	1283	5.6	**Ref**			**Ref**		
8 < 9	16,766	932	5.6	**1.01**	0.93	1.09	**0.99**	0.91	1.08
9 < 10	6859	373	5.4	**1.00**	0.90	1.12	**0.98**	0.87	1.09
10<	2974	141	4.7	**0.95**	0.80	1.12	**0.93**	0.79	1.10
Bedtime									
21 < 24	45,951	2623	5.7	**Ref**			**Ref**		
24 < 27	19,811	1053	5.3	**0.96**	0.90	1.03	**1.00**	0.93	1.07
Other	1804	86	4.8	**0.92**	0.74	1.13	**0.94**	0.76	1.16
**Problems solving**									
Sleep duration									
< 6 h	4482	246	5.5	**1.09**	0.95	1.25	**1.06**	0.93	1.21
6 < 7	13,404	726	5.4	**1.08**	0.98	1.18	**1.04**	0.95	1.14
7 < 8	23,041	1151	5.0	**Ref**			**Ref**		
8 < 9	16,757	831	5.0	**1.00**	0.92	1.09	**1.05**	0.96	1.15
9 < 10	6848	343	5.0	**1.03**	0.91	1.16	**1.12**	1.00	1.26
10 <	2973	141	4.7	**1.04**	0.88	1.24	**1.10**	0.93	1.31
Bedtime									
21 < 24	45,918	2321	5.1	**Ref**			**Ref**		
24 < 27	19,785	1042	5.3	**1.08**	1.00	1.16	**1.00**	0.93	1.08
Other	1802	75	4.2	**0.89**	0.71	1.12	**0.90**	0.72	1.13
**Personal-social characteristics**									
Sleep duration									
< 6 h	4480	50	1.1	**1.11**	0.82	1.51	**1.20**	0.88	1.62
6 < 7	13,402	142	1.1	**1.05**	0.85	1.29	**1.12**	0.91	1.37
7 < 8	23,017	231	1.0	**Ref**			**Ref**		
8 < 9	16,731	222	1.3	**1.33**	1.11	1.60	**1.23**	1.02	1.48
9 < 10	6852	99	1.4	**1.48**	1.17	1.87	**1.30**	1.03	1.65
10<	2971	34	1.1	**1.27**	0.89	1.82	**1.18**	0.82	1.69
Bedtime									
21 < 24	45,874	554	1.2	**Ref**			**Ref**		
24 < 27	19,778	211	1.1	**0.91**	0.78	1.07	**1.10**	0.93	1.29
Other	1801	13	0.7	**0.66**	0.38	1.14	**0.72**	0.41	1.24
**Total (abnormal score for any 1 of the 5 domain)**									
Sleep duration									
< 6 h	4491	621	13.8	**1.01**	0.94	1.10	**1.01**	0.93	1.09
6 < 7	13,432	1917	14.3	**1.04**	0.99	1.10	**1.03**	0.98	1.08
7 < 8	23,070	3146	13.6	**Ref**			**Ref**		
8 < 9	16,783	2218	13.2	**0.98**	0.93	1.03	**0.99**	0.95	1.05
9 < 10	6865	874	12.7	**0.96**	0.89	1.03	**0.99**	0.92	1.06
10<	2976	365	12.3	**1.00**	0.90	1.10	**1.02**	0.92	1.13
Bedtime									
21 < 24	45,987	6285	13.7	**Ref**			**Ref**		
24 < 27	19,823	2645	13.3	**1.01**	0.97	1.05	**0.98**	0.94	1.03
Other	1807	211	11.7	**0.94**	0.82	1.07	**0.97**	0.85	1.10

*CI* confidence interval, *RR* risk ratio. **Bold fonts** showed the items of the Ages and Stages Questionnaire.

^a^Adjusted for maternal age at delivery, smoking habits, alcohol consumption, pre-pregnancy body mass index, gestational age at birth, parity, infertility treatment, and infant sex.

**Table 5:** Association between sleep during pregnancy and infant development, Japan Environment and Children's Study (2011–2014).

	No. of participants	No. of outcome		Maternal age adjusted model			Multivariable model^a^		
			%	RR	95% CI		RR	95% CI	
**Communication**									
Sleep duration									
< 6 h	3186	5	0.2	**1.32**	0.51	3.43	**1.29**	0.49	3.35
6 < 7	10,130	12	0.1	**0.97**	0.49	1.93	**0.93**	0.47	1.86
7 < 8	21,181	26	0.1	**Ref**			**Ref**		
8 < 9	19,275	13	0.1	**0.55**	0.28	1.07	**0.60**	0.30	1.16
9 < 10	9528	10	0.1	**0.88**	0.42	1.83	**0.99**	0.47	2.08
10<	4291	1	0.0	**0.22**	0.03	1.65	**0.24**	0.03	1.80
Bedtime									
21 < 24	50,034	48	0.1	**Ref**			**Ref**		
24 < 27	16,083	18	0.1	**1.23**	0.72	2.12	**1.07**	0.61	1.86
Other	1474	1	0.1	**0.78**	0.11	5.64	**0.82**	0.11	5.95
Depth of sleep									
Very light	4563	4	0.1	**0.81**	0.28	2.30	**0.82**	0.29	2.35
Light	28,285	24	0.1	**0.78**	0.45	1.33	**0.79**	0.46	1.35
Normal	27,044	29	0.1	**Ref**			**Ref**		
Deep	6465	6	0.1	**0.88**	0.37	2.12	**0.86**	0.36	2.06
Very deep	1234	4	0.3	**3.16**	1.11	8.98	**3.01**	1.06	8.55
Feeling when waking up in the morning									
Very bad	991	3	0.3	**3.41**	1.06	10.99	**3.38**	1.05	10.88
Bad	13,634	15	0.1	**1.13**	0.63	2.04	**1.12**	0.62	2.02
Normal	42,197	42	0.1	**Ref**			**Ref**		
Good	9530	4	0.0	**0.41**	0.15	1.16	**0.40**	0.14	1.11
Very good	1239	3	0.2	**2.41**	0.75	7.75	**2.27**	0.70	7.33
**Gross motor skills**									
Sleep duration									
< 6 h	3186	191	6.0	**1.06**	0.92	1.23	**1.06**	0.92	1.23
6 < 7	10,130	613	6.1	**1.06**	0.97	1.17	**1.05**	0.95	1.15
7 < 8	21,184	1203	5.7	**Ref**			**Ref**		
8 < 9	19,275	1020	5.3	**0.94**	0.86	1.02	**0.96**	0.89	1.04
9 < 10	9526	497	5.2	**0.94**	0.85	1.04	**0.99**	0.89	1.09
10<	4292	186	4.3	**0.85**	0.73	0.99	**0.88**	0.76	1.03
Bedtime									
21 < 24	50,037	2778	5.6	**Ref**			**Ref**		
24 < 27	16,079	842	5.2	**0.98**	0.91	1.05	**0.93**	0.86	1.01
Other	1477	90	6.1	**1.16**	0.95	1.43	**1.19**	0.97	1.46
Depth of sleep									
Very light	4564	258	5.7	**1.00**	0.88	1.13	**1.02**	0.90	1.16
Light	28,288	1521	5.4	**0.95**	0.89	1.02	**0.96**	0.89	1.03
Normal	27,045	1512	5.6	**Ref**			**Ref**		
Deep	6463	355	5.5	**0.99**	0.89	1.11	**0.99**	0.88	1.10
Very deep	1233	64	5.2	**0.95**	0.75	1.21	**0.96**	0.75	1.22
Feeling when waking up in the morning									
Very bad	991	48	4.8	**0.95**	0.72	1.25	**0.97**	0.73	1.28
Bad	13,636	762	5.6	**1.04**	0.96	1.13	**1.05**	0.97	1.13
Normal	42,197	2306	5.5	**Ref**			**Ref**		
Good	9531	529	5.6	**1.00**	0.92	1.10	**1.00**	0.91	1.09
Very good	1238	65	5.3	**0.95**	0.75	1.21	**0.93**	0.73	1.17
**Fine motor skills**									
Sleep duration									
< 6 h	3184	180	5.7	**1.00**	0.85	1.16	**1.00**	0.86	1.16
6 < 7	10,124	587	5.8	**1.01**	0.92	1.11	**1.03**	0.93	1.13
7 < 8	21,178	1210	5.7	**Ref**			**Ref**		
8 < 9	19,265	1068	5.5	**0.97**	0.90	1.06	**0.96**	0.89	1.04
9 < 10	9524	492	5.2	**0.93**	0.84	1.02	**0.90**	0.82	1.00
10<	4291	225	5.2	**1.01**	0.88	1.16	**1.01**	0.88	1.16
Bedtime									
21 < 24	50,017	2841	5.7	**Ref**			**Ref**		
24 < 27	16,074	838	5.2	**0.95**	0.88	1.02	**0.97**	0.90	1.05
Other	1475	83	5.6	**1.04**	0.84	1.29	**1.05**	0.85	1.29
Depth of sleep									
Very light	4558	251	5.5	**0.96**	0.84	1.09	**0.94**	0.82	1.06
Light	28,280	1577	5.6	**0.97**	0.91	1.04	**0.97**	0.90	1.04
Normal	27,035	1532	5.7	**Ref**			**Ref**		
Deep	6462	351	5.4	**0.97**	0.87	1.08	**0.98**	0.88	1.10
Very deep	1231	51	4.1	**0.75**	0.57	0.98	**0.76**	0.58	1.00
Feeling when waking up in the morning									
Very bad	990	58	5.9	**1.09**	0.85	1.41	**1.08**	0.84	1.39
Bad	13,635	829	6.1	**1.09**	1.01	1.17	**1.08**	1.00	1.17
Normal	42,175	2396	5.7	**Ref**			**Ref**		
Good	9528	425	4.5	**0.78**	0.70	0.86	**0.78**	0.71	0.86
Very good	1238	54	4.4	**0.76**	0.58	0.99	**0.78**	0.60	1.01
**Problems solving**									
Sleep duration									
< 6 h	3182	182	5.7	**1.09**	0.94	1.27	**1.07**	0.92	1.25
6 < 7	10,115	525	5.2	**0.99**	0.89	1.09	**0.96**	0.87	1.07
7 < 8	21,161	1105	5.2	**Ref**			**Ref**		
8 < 9	19,253	970	5.0	**0.97**	0.89	1.05	**1.01**	0.93	1.10
9 < 10	9508	438	4.6	**0.90**	0.81	1.01	**0.96**	0.86	1.07
10<	4286	218	5.1	**1.07**	0.92	1.23	**1.12**	0.97	1.29
Bedtime									
21 < 24	49,976	2544	5.1	**Ref**			**Ref**		
24 < 27	16,057	817	5.1	**1.03**	0.96	1.11	**0.96**	0.89	1.04
Other	1472	77	5.2	**1.08**	0.86	1.34	**1.09**	0.88	1.36
Depth of sleep									
Very light	4557	211	4.6	**0.84**	0.73	0.97	**0.85**	0.74	0.98
Light	28,256	1406	5.0	**0.91**	0.85	0.97	**0.92**	0.86	0.99
Normal	27,001	1463	5.4	**Ref**			**Ref**		
Deep	6458	312	4.8	**0.90**	0.80	1.02	**0.89**	0.79	1.01
Very deep	1233	46	3.7	**0.70**	0.53	0.94	**0.70**	0.53	0.93
Feeling when waking up in the morning									
Very bad	990	63	6.4	**1.28**	1.01	1.64	**1.29**	1.01	1.64
Bad	13,620	737	5.4	**1.05**	0.97	1.14	**1.05**	0.97	1.14
Normal	42,133	2204	5.2	**Ref**			**Ref**		
Good	9525	390	4.1	**0.77**	0.70	0.86	**0.77**	0.69	0.85
Very good	1237	44	3.6	**0.67**	0.50	0.90	**0.66**	0.50	0.89
**Personal-social characteristics**									
Sleep duration									
< 6 h	3182	40	1.3	**1.27**	0.91	1.77	**1.33**	0.95	1.85
6 < 7	10,110	105	1.0	**1.04**	0.82	1.31	**1.10**	0.87	1.38
7 < 8	21,141	211	1.0	**Ref**			**Ref**		
8 < 9	19,222	259	1.4	**1.36**	1.13	1.63	**1.27**	1.06	1.53
9 < 10	9511	112	1.2	**1.21**	0.96	1.52	**1.08**	0.86	1.36
10<	4287	51	1.2	**1.31**	0.97	1.78	**1.24**	0.92	1.69
Bedtime									
21 < 24	49,936	584	1.2	**Ref**			**Ref**		
24 < 27	16,045	173	1.1	**0.95**	0.80	1.13	**1.12**	0.94	1.33
Other	1472	21	1.4	**1.29**	0.83	1.98	**1.29**	0.84	1.99
Depth of sleep									
Very light	4553	57	1.3	**1.01**	0.76	1.33	**0.95**	0.72	1.26
Light	28,224	318	1.1	**0.91**	0.78	1.06	**0.89**	0.76	1.03
Normal	26,991	331	1.2	**Ref**			**Ref**		
Deep	6451	59	0.9	**0.75**	0.57	0.99	**0.79**	0.60	1.04
Very deep	1234	13	1.1	**0.88**	0.51	1.53	**0.92**	0.53	1.60
Feeling when waking up in the morning									
Very bad	991	17	1.7	**1.59**	0.99	2.57	**1.58**	0.98	2.55
Bad	13,608	179	1.3	**1.17**	0.98	1.38	**1.16**	0.98	1.38
Normal	42,104	482	1.1	**Ref**			**Ref**		
Good	9514	82	0.9	**0.75**	0.59	0.94	**0.76**	0.60	0.96
Very good	1236	18	1.5	**1.26**	0.79	2.01	**1.32**	0.82	2.10
**Total (abnormal score for any 1 of the 5 domain)**									
Sleep duration									
< 6 h	3187	460	14.4	**1.04**	0.95	1.14	**1.04**	0.95	1.13
6 < 7	10,132	1443	14.2	**1.02**	0.96	1.08	**1.01**	0.95	1.07
7 < 8	21,191	2957	14.0	**Ref**			**Ref**		
8 < 9	19,283	2547	13.2	**0.95**	0.90	1.00	**0.97**	0.92	1.02
9 < 10	9531	1217	12.8	**0.93**	0.88	0.99	**0.96**	0.90	1.02
10<	4293	517	12.0	**0.95**	0.87	1.03	**0.98**	0.89	1.07
Bedtime									
21 < 24	50,054	6822	13.6	**Ref**			**Ref**		
24 < 27	16,086	2118	13.2	**1.00**	0.95	1.04	**0.97**	0.93	1.02
Other	1477	201	13.6	**1.05**	0.92	1.20	**1.07**	0.94	1.22
Depth of sleep									
Very light	4564	601	13.2	**0.93**	0.86	1.01	**0.94**	0.87	1.02
Light	28,297	3794	13.4	**0.95**	0.91	0.99	**0.96**	0.92	1.00
Normal	27,054	3762	13.9	**Ref**			**Ref**		
Deep	6468	845	13.1	**0.95**	0.89	1.02	**0.95**	0.88	1.02
Very deep	1234	139	11.3	**0.83**	0.70	0.97	**0.83**	0.71	0.98
Feeling when waking up in the morning									
Very bad	991	133	13.4	**1.04**	0.89	1.22	**1.05**	0.89	1.23
Bad	13,641	1947	14.3	**1.06**	1.01	1.11	**1.06**	1.01	1.11
Normal	42,211	5779	13.7	**Ref**			**Ref**		
Good	9535	1143	12.0	**0.87**	0.82	0.92	**0.86**	0.81	0.91
Very good	1239	139	11.2	**0.81**	0.69	0.95	**0.81**	0.69	0.95

*CI* confidence interval, *RR* risk ratio.  **Bold fonts** showed the items of the Ages and Stages Questionnaire.

^a^Adjusted for maternal age at delivery, smoking habits, alcohol consumption, pre-pregnancy body mass index, gestational age at birth, parity, infertility treatment, and infant sex.

## Discussion

This study investigated whether maternal sleep before and during pregnancy was associated with sleeping or developmental problems in 1-year-old infants, using data from a nationwide large-scale cohort study in Japan. The present study showed that maternal short or long sleep and bedtime after midnight, both before and during pregnancy, increased the risk ratio of the offspring’s sleeping problems at 1 year of age. The sub-analysis limited to participants with adequate sleep durations showed that maternal sleep pattern both before and during pregnancy was associated with the infants’ sleep outcomes. In addition, maternal subjective deep sleep and good mood at waking up during pregnancy were inversely associated with the infants’ sleep problems and the J-ASQ abnormal scores.

In this study, the participants tended to sleep longer and go to bed earlier during pregnancy than they did before pregnancy. In Japan, many women still stop working due to pregnancy or take maternity leave during late pregnancy. For that reason, sleep duration and bedtime might improve during pregnancy.

Sleep cycle develops from the fetal period^13^. Animal studies have shown that the circadian rhythm is affected by maternal life rhythms via endogenous substances such as melatonin^14,15^. Animal studies have also reported that exposure to sleep deprivation or artificial disappearance of light–dark cycle during pregnancy affects the offspring’s circadian rhythm abnormality and abnormal behavioral pattern^16,17^. In this study, mother’s short sleep and late bedtime were associated with the offspring’s sleeping problems, in part, because of the influence of maternal life rhythm during the fetal period.

In addition, it is considered that postpartum sleep pattern would partly correlate with sleep pattern before or during pregnancy. The study of 18-month-old twin infants reported that the genetic effect on sleep duration was 30.8% and the environmental effect was 64.1%^18^. The association between sleep before or during pregnancy and infant sleeping problems may be influenced via life rhythm after childbirth.

Subjective sleep quality was associated not only with infants’ sleep problem but also with the risk of abnormal ASQ scores. Subjective light sleep and bad mood upon wakening may reflect maternal SDB or depression^19,20^. Furthermore, it has been reported that both of these factors are related to the offspring’s development^10,21^. One potential factor explaining the association between maternal sleep and the offspring’s outcomes is inflammation. SDB and maternal depression increase inflammatory cytokine levels^22–24^; maternal inflammation during pregnancy can cause developmental disorders^25,26^. In addition, maternal SDB may affect the offspring’s development via low birth weight, which has been reported to be associated with neurodevelopment^27,28^. Interventions in maternal SDB and depression during pregnancy may improve subjective sleep quality and subsequent offspring sleep and development.

We have previously reported that maternal sleep habits, such as short sleep and late bedtime, before and during pregnancy, increased the risk ratio of long sleep duration during the day, bad mood, frequency of crying for a long time, and intense crying in 1-month-old offspring^12^. We further showed the association between maternal unsuitable sleep habits and the offspring’s non-desirable sleep habits as lasting even 1 year after birth. It is expected that children’s sleep and development will be influenced more by factors after birth than by prenatal ones. Therefore, it is important to clarify how long maternal sleep habits both before and during pregnancy are related to offspring’s sleep and developmental progression and to verify whether an intervention of maternal sleep, at any time point, improves offspring’s sleep and developmental outcomes.

This study was not without limitations. Because the present study was an observational study, confounding factors, such as parental life rhythm, that were not part of our evaluations might have been present. Moreover, information regarding both maternal sleep habits and infant’s outcomes was collected using a self-reported questionnaire, and thus, it had a risk of bias, such as a recall bias. The questions about maternal and infant sleep have not been previously validated. For example, we used the frequency of infant’s night crying as outcome, but we could not get the intended information about the duration and reason for the infant crying. Thus, there could be some bias, such as reporting bias. Additionally, about 13% of the participants were excluded from the analysis due to lack of information about exposure, covariates, and outcomes, and this group tended to be younger and with more smokers than the mothers who responded. This may be an added bias. In addition, because each association between maternal sleep and outcomes was tested separately, multiple testing may be a limitation. However, a strong point of this study is that our results were derived from large-scale nationwide data. To the best of our knowledge, there is no other study of this size on how maternal sleep during pregnancy correlates with offspring sleep behavior.

In conclusion, maternal short or long sleep duration and late bedtime, both before and during pregnancy, may increase sleeping problems such as late bedtime, awakening during night, and short sleep in 1-year-old offspring. Additionally, subjective maternal deep sleep and good mood at waking up during pregnancy decreased the risk ratio of infants’ sleeping problem and the ASQ abnormal scores.

## Methods

### Research ethics

The study protocol was approved by the Ministry of Environment’s Institutional Review Board on Epidemiological Studies and by the Ethics Committee of all participating institutions: the National Institute for Environmental Studies that leads the Japan Environment and Children’s Study (JECS), the National Center for Child Health and Development, Hokkaido University, Sapporo Medical University, Asahikawa Medical College, Japanese Red Cross Hokkaido College of Nursing, Tohoku University, Fukushima Medical University, Chiba University, Yokohama City University, University of Yamanashi, Shinshu University, University of Toyama, Nagoya City University, Kyoto University, Doshisha University, Osaka University, Osaka Medical Center and Research Institute for Maternal and Child Health, Hyogo College of Medicine, Tottori University, Kochi University, University of Occupational and Environmental Health, Kyushu University, Kumamoto University, University of Miyazaki, and University of Ryukyu. Written informed consent was obtained from all participants. All methods were performed in accordance with the approved guidelines.

### Study participants

The data used in this study were obtained from the JECS, an ongoing large-scale cohort study. The JECS elucidated environmental factors that are associated with children’s health and development, and was designed to follow women through their pregnancy until their newborns grow up to be 13 years old. The participants were recruited between 2011 and 2014 from 15 regions throughout Japan, and the follow-up was mainly conducted via a self-administered questionnaire. The detailed protocol has been reported elsewhere^29^. The baseline profiles of participants of the JECS have been reported previously^30^. Participants answered a questionnaire about lifestyle and behavior twice during pregnancy. The questionnaire answered at recruitment was M-T1 and answered later during mid and late pregnancy was M-T2. The mean gestational weeks (SD, 5–95 percentile) at the time of answering M-T1 and M-T2 were 16.4 (8.0, 9–29) and 27.9 (6.5, 25–35) weeks, respectively. Participants also answered a questionnaire about their offspring 1 year after delivery (C-1y).

We excluded cases of multiple pregnancies (n = 949), preterm or post-term deliveries (before 37 weeks or after 42 weeks of gestation) (n = 4184), and congenital anomalies identified before 1 month of age (n = 3553). These factors are thought to be associated with infant development. For women who participated in the JECS study multiple times, data from the second and subsequent participations were excluded (n = 5647). In addition, we excluded cases for which information required for analysis was not available: miscarriage or stillbirth (n = 3676), missing information on maternal age at delivery (n = 7), lack of information about covariates (n = 450), incomplete information on maternal sleep at both M-T1 and M-T2 (n = 3376), missing responses to all questions about children’s sleep habits and developmental progress at C-1y (n = 7393).

**Figure 1:**
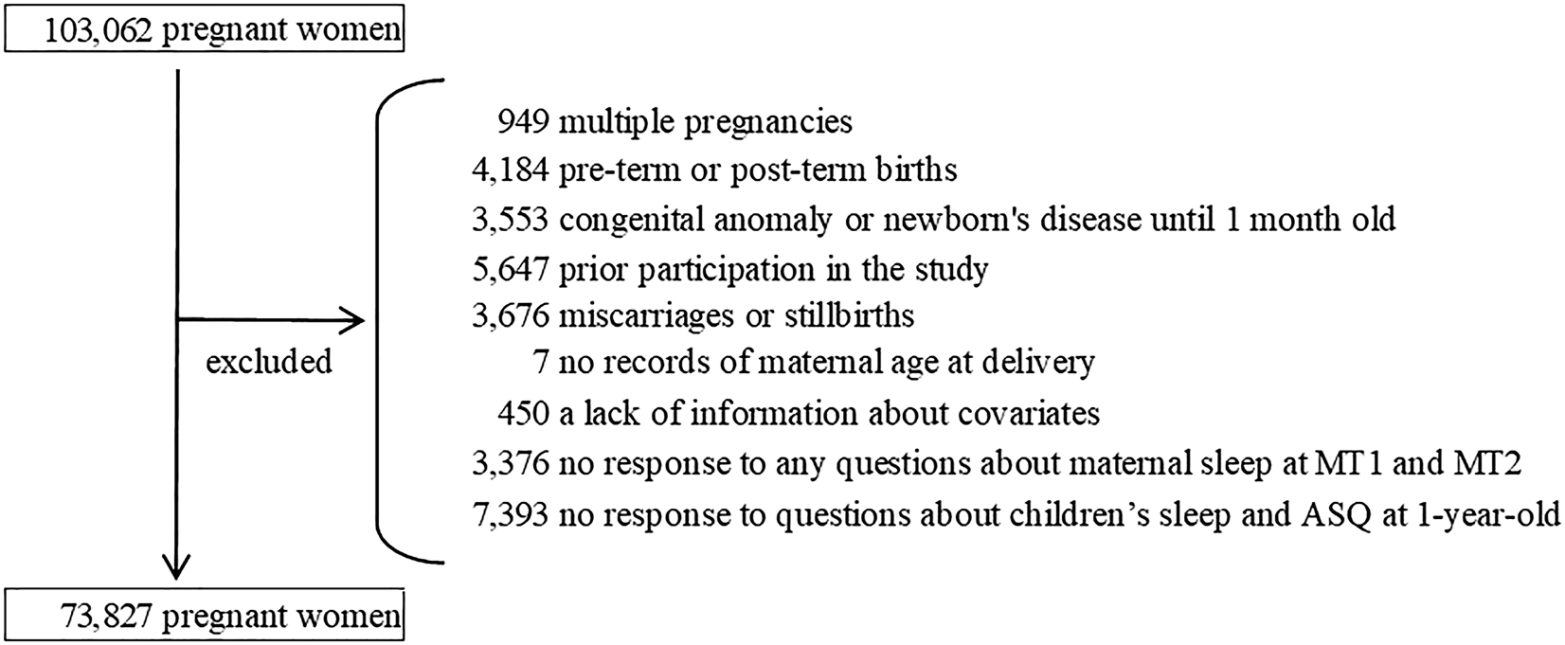
Flow chart representing the study population. *MT1* questionnaire administered at recruitment, *MT2* questionnaire administered during mid- or late-pregnancy, *ASQ* the Ages and Stages Questionnaire.

The remaining 73,827 participants were included in the analysis (Fig. 1). To determine the risk of potential bias due to missing data, we compared the background characteristics between the population analyzed and the population excluded from analysis due to a lack of information about covariates and non-response to any questions about maternal sleep or children’s sleep and development (Supplemental Table 3). The group excluded from the analysis had more participants who were less than 25 years old and had smoking habits, lower educational background, and lower household income.

### Maternal sleep

The categorization of maternal sleep was done as in our previous research^12^.

In the M-T1 questionnaire, participants were asked about their awakening time and bedtime before pregnancy. We calculated the sleep duration of participants and divided the participants into six groups according to sleep time: < 6 h, 6–7 h, 7–8 h (reference), 8–9 h, 9–10 h, and > 10 h. Participants were also divided by bedtime: 9.00 p.m. to midnight (reference), midnight to 3.00 a.m., and others (sleep before 9.00 p.m. or after 3.00 a.m.). The bedtime for more than 95% of the analyzed subjects was between 21:00 and 27:00. Since the mode of bedtime was between 22:00 and 24:00, we further divided the participants by bedtime 24:00.

In the M-T2 questionnaire, participants were asked about their usual awakening time and bedtime in the last month. The participants were divided into groups as described above for M-T1. Furthermore, the M-T2 questionnaire included two additional questions regarding sleep quality. One was “How would you rate your average depth of sleep during the past month?” The other one was “How would you rate your overall feeling when waking up in the morning, during the past month?” The answers to both questions were scored on a 1–5 scale, representing very light/bad, relatively light/bad, normal (reference), relatively deep/good, and very deep/good, respectively. Both of these questionnaires (M-T1 and M-T2) have not been previously validated.

### Outcome 1: offspring’s sleeping problems

One year after delivery, information on infant sleep habits and crying at night was collected via a parent-reported questionnaire (C-1y). The participants answered their infant sleep time in the last 24 h in 30-min increments. They were also asked whether their children cried at night over the last month, and if so, the frequency (“rarely”, “1–3 times in a month”, “1–2 times in a week”, “3–4 times in a week”, “5 times in a week or more”). The questionnaires used for this outcome have not been previously validated. In this analysis, we focused on five points. First, from the responses regarding the infant’s sleep the day before, we determined the number of nocturnal awakenings. A previous study reported that the upper limit of the number of awakenings during the night is 2.5 for 1-year-old infants^31^; as such, we defined ≥ 3 awakenings as too many. Second, we analyzed whether the infants awoke more than once and whether they stayed awake for more than 1 h during the night. Third, we analyzed the duration of nocturnal sleep (from 20:00 to 07:59). We regarded less than 8 h of sleep as unusual. Fourth, we collected information regarding the infants’ bedtime. Based on previous studies^32,33^, we defined bedtime after 22:00 as too late. Fifth, we analyzed nocturnal crying frequency during the past month. If the mother answered that her infant awoke and cried during the night, and that the frequency of crying at night was more than five times per week, we defined the case as “crying at night”.

### Offspring’s development

We used the J-ASQ-3 to evaluate offspring’s development. The C-1y questionnaire included a J-ASQ-3 assessment. J-ASQ-3 captures any developmental delay in five domains: communication, gross motor skills, fine motor skills, problem solving, and personal–social characteristics. The answer to each question is one of the following: “yes,” “sometimes,” or “not yet.” Scores are 10, 5, and 0 points, respectively. Each J-ASQ-3 domain was composed of six questions, and the total score ranged from 0 to 60. Higher scores were defined as more developed, and the cutoff points for every domain in the Japanese version were determined by a previous study^34^. We defined outcomes by whether the score was less than the determined cut-off point of each J-ASQ-3 domain and whether the score was less than the cutoff point of any one of the five J-ASQ-3 domains.

### Covariates

Information about maternal age at delivery, smoking habits, alcohol consumption, pre-pregnancy body mass index (BMI), parity, gestational age at birth, infertility treatment, and infant sex, was collected via self-administered questionnaires and/or medical records. These selected covariates were reported as risk factors for developmental disorders^35–37^.

### Statistical analyses

We used a log-binominal regression model to explore the association of maternal sleep with each outcome and to estimate the RRs of each outcome and 95% CIs. We initially adjusted for maternal age at delivery and then further adjusted for smoking habits (never smoked, ex-smokers who quit before pregnancy, smokers during early pregnancy), alcohol consumption (never drinkers, ex-drinkers who quit before pregnancy, drinkers during early pregnancy), pre-pregnancy BMI (< 18.5, 18.5–24.9, ≥ 25.0 kg/m^2^), parity (0, ≥ 1), infertility treatment (no ovulation stimulation/artificial insemination by sperm from husband, assisted reproductive technology), gestational age at birth (37–38, 39–41 weeks), and infant sex (boys, girls). In this study, we did not actively complete any missing data, and all analysis was limited to data from those participants who provided complete information for exposures, outcomes, and covariates. In addition, we performed a sub-analysis twice to evaluate which maternal sleep, the one before or one during pregnancy, impacts the infant’s sleep outcome. In the first sub-analysis, we limited it to the participant groups with adequate sleep duration of 7–9 h during pregnancy and investigated the association between maternal sleep before pregnancy and infant’s sleep. We limited the second analysis to the participant groups with sleep duration of 7–9 h before pregnancy and examined the association between maternal sleep during pregnancy and infant’s sleep.

These statistical analyses were almost the same as those used in our previous study^12^.

In this study, we used a fixed dataset “jecs-an-20180131,” which was released in March 2018. Stata version 15 (StataCorp LP, College Station, TX, USA) was used for all analyses.

## Supplementary Information

Supplementary Information.
