# Supplementary material for: Association of maternal sleep before and during pregnancy with sleep and developmental problems in 1-year-old infants
Source: Sci Rep. 2021 Jun 4;11:11834. doi: 10.1038/s41598-021-91271-7 (PMC8178306; doi:10.1038/s41598-021-91271-7)
Supplement: Supplementary file 1 — Supplementary Information. [file 41598_2021_91271_MOESM1_ESM.docx]

**Supplemental tables**

**Association of maternal sleep before and during pregnancy with sleep and developmental problems in 1-year-old infants**

Kazushige Nakahara, Takehiro Michikawa, Seiichi Morokuma, Masanobu Ogawa, Kiyoko Kato, Masafumi Sanefuji, Eiji Shibata, Mayumi Tsuji, Masayuki Shimono, Toshihiro Kawamoto, Shouichi Ohga, Koichi Kusuhara, and the Japan Environment and Children’s Study Group

| Supplemental Table 1. Baseline characteristics of the study population stratified by sleep duration during pregnancy | | | | | | | | | | | | |  |  |
| --- | --- | --- | --- | --- | --- | --- | --- | --- | --- | --- | --- | --- | --- | --- |
|  |  |  |  |  |  |  |  |  |  |  |  |  |  |  |
|  |  |  | Maternal sleep during pregnancy | | | | | | | | | | | |
|  |  |  | <6 hours | | 6<7 | | 7<8 | | 8<9 | | 9<10 | | 10< | |
|  |  |  | n | % | n | % | n | % | n | % | n | % | n | % |
| Maternal sleep | | | 3,540 | 4.8 | 11,099 | 15.0 | 23,050 | 31.2 | 21,043 | 28.5 | 10,394 | 14.1 | 4,701 | 6.4 |
|  |  |  |  |  |  |  |  |  |  |  |  |  |  |  |
| Maternal characteristics | | |  |  |  |  |  |  |  |  |  |  |  |  |
|  | Age at delivery (years) | |  |  |  |  |  |  |  |  |  |  |  |  |
|  |  | < 25 | 367 | 10.4 | 857 | 7.7 | 1,659 | 7.2 | 1,600 | 7.6 | 1,041 | 10.0 | 968 | 20.6 |
|  |  | 25–29 | 903 | 25.5 | 2,963 | 26.7 | 6,290 | 27.3 | 5,785 | 27.5 | 2,948 | 28.4 | 1,524 | 32.4 |
|  |  | 30–34 | 1,152 | 32.5 | 3,835 | 34.6 | 8,491 | 36.8 | 7,840 | 37.3 | 3,881 | 37.3 | 1,388 | 29.5 |
|  |  | ≥ 35 | 1,118 | 31.6 | 3,444 | 31.0 | 6,610 | 28.7 | 5,818 | 27.7 | 2,524 | 24.3 | 821 | 17.5 |
|  | Smoking habits | |  |  |  |  |  |  |  |  |  |  |  |  |
|  |  | Never smoked | 1,963 | 55.5 | 6,774 | 61.0 | 14,179 | 61.5 | 12,694 | 60.3 | 6,043 | 58.1 | 2,463 | 52.4 |
|  |  | Ex-smokers who quit before pregnancy | 771 | 21.8 | 2,419 | 21.8 | 5,255 | 22.8 | 5,089 | 24.2 | 2,656 | 25.6 | 1,174 | 25.0 |
|  |  | Smokers during early pregnancy | 806 | 22.8 | 1,906 | 17.2 | 3,616 | 15.7 | 3,260 | 15.5 | 1,695 | 16.3 | 1,064 | 22.6 |
|  | Alcohol consumption | |  |  |  |  |  |  |  |  |  |  |  |  |
|  |  | Never drank | 1,186 | 33.5 | 3,721 | 33.5 | 7,906 | 34.3 | 7,350 | 34.9 | 3,681 | 35.4 | 1,650 | 35.1 |
|  |  | Ex-drinkers who quit before pregnancy | 602 | 17.0 | 1,773 | 16.0 | 3,997 | 17.3 | 3,850 | 18.3 | 2,111 | 20.3 | 993 | 21.1 |
|  |  | Drinkers during early pregnancy | 1,752 | 49.5 | 5,605 | 50.5 | 11,147 | 48.4 | 9,843 | 46.8 | 4,602 | 44.3 | 2,058 | 43.8 |
|  | Pre-pregnancy body mass index (kg/m^2^) | |  |  |  |  |  |  |  |  |  |  |  |  |
|  |  | <18.5 | 588 | 16.6 | 1,756 | 15.8 | 3,682 | 16.0 | 3,299 | 15.7 | 1,735 | 16.7 | 825 | 17.6 |
|  |  | 18.5–24.9 | 2,564 | 72.4 | 8,261 | 74.4 | 17,173 | 74.5 | 15,662 | 74.4 | 7,590 | 73.0 | 3,385 | 72.0 |
|  |  | ≥ 25.0 | 388 | 11.0 | 1,082 | 9.8 | 2,195 | 9.5 | 2,082 | 9.9 | 1,069 | 10.3 | 491 | 10.4 |
|  | Parity | |  |  |  |  |  |  |  |  |  |  |  |  |
|  |  | 0 | 2,016 | 57.0 | 6,509 | 58.6 | 11,506 | 49.9 | 7,751 | 36.8 | 2,991 | 28.8 | 2,052 | 43.7 |
|  |  | ≥ 1 | 1,524 | 43.1 | 4,590 | 41.4 | 11,544 | 50.1 | 13,292 | 63.2 | 7,403 | 71.2 | 2,649 | 56.4 |
|  | Infertility treatment | |  |  |  |  |  |  |  |  |  |  |  |  |
|  |  | No | 3,292 | 93.0 | 10,144 | 91.4 | 21,332 | 92.6 | 19,698 | 93.6 | 9,879 | 95.1 | 4,492 | 95.6 |
|  |  | Ovulation stimulation/artificial insemination by sperm from husband | 140 | 4.0 | 501 | 4.5 | 931 | 4.0 | 749 | 3.6 | 302 | 2.9 | 127 | 2.7 |
|  |  | Assisted reproductive technology | 108 | 3.1 | 454 | 4.1 | 787 | 3.4 | 596 | 2.8 | 213 | 2.1 | 82 | 1.7 |
|  | Gestational age (weeks) | |  |  |  |  |  |  |  |  |  |  |  |  |
|  |  | Early term (37–38) | 1,140 | 32.2 | 3,495 | 31.5 | 7,462 | 32.4 | 6,963 | 33.1 | 3,510 | 33.8 | 1,482 | 31.5 |
|  |  | Full term (39–41) | 2,400 | 67.8 | 7,604 | 68.5 | 15,588 | 67.6 | 14,080 | 66.9 | 6,884 | 66.2 | 3,219 | 68.5 |
| Infant sex | | |  |  |  |  |  |  |  |  |  |  |  |  |
|  |  | Boys | 1,808 | 51.1 | 5,617 | 50.6 | 11,709 | 50.8 | 10,656 | 50.6 | 5,396 | 51.9 | 2,402 | 51.1 |
|  |  | Girls | 1,732 | 48.9 | 5,482 | 49.4 | 11,341 | 49.2 | 10,387 | 49.4 | 4,998 | 48.1 | 2,299 | 48.9 |

| Supplemental Table 2. Association between maternal sleep duration before or during pregnancy and infant sleep: sub-analysis of the Japan Environment and Children’s Study (2011–2014) | | | | | | | | | | | | | | | |
| --- | --- | --- | --- | --- | --- | --- | --- | --- | --- | --- | --- | --- | --- | --- | --- |
|  |  |  |  | **Sleep before pregnancy in participants** | | | | |  |  | **Sleep during pregnancy in participants** | | | | |
|  |  |  |  | **sleeping for 7 to 9 hours during pregnancy** | | | | |  |  | **sleeping for 7 to 9 hours before pregnancy** | | | | |
|  |  |  | **No. of**  **participants** | **No. of outcome** | | **Multivariable model ^a^** | | |  | **No. of participants** | **No. of outcome** | | **Multivariable model ^a^** | | |
|  |  |  |  |  | **%** | **RR** | **95% CI** | |  |  |  | **%** | **RR** | **95% CI** | |
| **>3 nighttime waking instances** | | | |  |  |  |  |  |  |  |  |  |  |  |  |
|  | **Sleep duration** | |  |  |  |  |  |  |  |  |  |  |  |  |  |
|  |  | **<6 hours** | 1,407 | 36 | 2.6 | **1.21** | 0.86 | 1.69 |  | 604 | 13 | 2.2 | **1.01** | 0.58 | 1.75 |
|  |  | **6<7** | 6,937 | 144 | 2.1 | **0.91** | 0.76 | 1.10 |  | 3,537 | 77 | 2.2 | **0.94** | 0.74 | 1.20 |
|  |  | **7<8** | 19,180 | 443 | 2.3 | **Ref** |  |  |  | 15,975 | 384 | 2.4 | **Ref** |  |  |
|  |  | **8<9** | 12,809 | 320 | 2.5 | **1.08** | 0.94 | 1.25 |  | 16,014 | 379 | 2.4 | **0.97** | 0.85 | 1.12 |
|  |  | **9<10** | 2,787 | 74 | 2.7 | **1.19** | 0.93 | 1.51 |  | 5,442 | 144 | 2.7 | **1.11** | 0.92 | 1.34 |
|  |  | **10<** | 973 | 22 | 2.3 | **1.12** | 0.73 | 1.71 |  | 1,866 | 53 | 2.8 | **1.31** | 0.98 | 1.74 |
|  | **Bedtime** | |  |  |  |  |  |  |  |  |  |  |  |  |  |
|  |  | **21<24** | 32,644 | 782 | 2.4 | **Ref** |  |  |  | 35,493 | 882 | 2.5 | **Ref** |  |  |
|  |  | **24<27** | 10,676 | 242 | 2.3 | **1.01** | 0.87 | 1.18 |  | 7,315 | 146 | 2.0 | **0.86** | 0.72 | 1.03 |
|  |  | **Other** | 773 | 15 | 1.9 | **0.94** | 0.57 | 1.57 |  | 630 | 22 | 3.5 | **1.53** | 1.01 | 2.32 |
|  |  |  |  |  |  |  |  |  |  |  |  |  |  |  |  |
| **>1 waking instances lasting >1 hour** | | | |  |  |  |  |  |  |  |  |  |  |  |  |
|  | **Sleep duration** | |  |  |  |  |  |  |  |  |  |  |  |  |  |
|  |  | **<6 hours** | 1,407 | 103 | 7.3 | **1.34** | 1.10 | 1.63 |  | 604 | 43 | 7.1 | **1.34** | 0.99 | 1.80 |
|  |  | **6<7** | 6,937 | 402 | 5.8 | **1.09** | 0.97 | 1.22 |  | 3,537 | 192 | 5.4 | **1.03** | 0.88 | 1.20 |
|  |  | **7<8** | 19,180 | 1,003 | 5.2 | **Ref** |  |  |  | 15,975 | 832 | 5.2 | **Ref** |  |  |
|  |  | **8<9** | 12,809 | 639 | 5.0 | **0.97** | 0.88 | 1.07 |  | 16,014 | 810 | 5.1 | **0.99** | 0.90 | 1.08 |
|  |  | **9<10** | 2,787 | 148 | 5.3 | **1.03** | 0.87 | 1.22 |  | 5,442 | 278 | 5.1 | **1.00** | 0.87 | 1.14 |
|  |  | **10<** | 973 | 78 | 8.0 | **1.47** | 1.18 | 1.84 |  | 1,866 | 104 | 5.6 | **1.07** | 0.87 | 1.30 |
|  | **Bedtime** | |  |  |  |  |  |  |  |  |  |  |  |  |  |
|  |  | **21<24** | 32,644 | 1,580 | 4.8 | **Ref** |  |  |  | 35,493 | 1,714 | 4.8 | **Ref** |  |  |
|  |  | **24<27** | 10,676 | 710 | 6.7 | **1.34** | 1.22 | 1.46 |  | 7,315 | 490 | 6.7 | **1.38** | 1.25 | 1.52 |
|  |  | **Other** | 773 | 83 | 10.7 | **2.13** | 1.73 | 2.64 |  | 630 | 55 | 8.7 | **1.80** | 1.39 | 2.32 |
|  |  |  |  |  |  |  |  |  |  |  |  |  |  |  |  |
| **<8 hours of sleep during the night (20:00–7:59)** | | | | |  |  |  |  |  |  |  |  |  |  |  |
|  | **Sleep duration** | |  |  |  |  |  |  |  |  |  |  |  |  |  |
|  |  | **<6 hours** | 1,407 | 95 | 6.8 | **1.38** | 1.12 | 1.69 |  | 604 | 42 | 7.0 | **1.40** | 1.04 | 1.89 |
|  |  | **6<7** | 6,937 | 364 | 5.3 | **1.09** | 0.97 | 1.23 |  | 3,537 | 213 | 6.0 | **1.21** | 1.05 | 1.41 |
|  |  | **7<8** | 19,180 | 911 | 4.8 | **Ref** |  |  |  | 15,975 | 789 | 4.9 | **Ref** |  |  |
|  |  | **8<9** | 12,809 | 531 | 4.2 | **0.88** | 0.80 | 0.98 |  | 16,014 | 653 | 4.1 | **0.83** | 0.75 | 0.92 |
|  |  | **9<10** | 2,787 | 124 | 4.5 | **0.95** | 0.79 | 1.15 |  | 5,442 | 251 | 4.6 | **0.95** | 0.83 | 1.09 |
|  |  | **10<** | 973 | 65 | 6.7 | **1.39** | 1.08 | 1.77 |  | 1,866 | 89 | 4.8 | **0.98** | 0.79 | 1.21 |
|  | **Bedtime** | |  |  |  |  |  |  |  |  |  |  |  |  |  |
|  |  | **21<24** | 32,644 | 1,437 | 4.4 | **Ref** |  |  |  | 35,493 | 1,576 | 4.4 | **Ref** |  |  |
|  |  | **24<27** | 10,676 | 581 | 5.4 | **1.22** | 1.11 | 1.35 |  | 7,315 | 413 | 5.7 | **1.27** | 1.14 | 1.42 |
|  |  | **Other** | 773 | 72 | 9.3 | **2.06** | 1.64 | 2.59 |  | 630 | 48 | 7.6 | **1.72** | 1.30 | 2.26 |
|  |  |  |  |  |  |  |  |  |  |  |  |  |  |  |  |
| **Falling asleep at 22:00 or later** | | | | | | |  |  |  |  |  |  |  |  |  |
|  | **Sleep duration** | |  |  |  |  |  |  |  |  |  |  |  |  |  |
|  |  | **<6 hours** | 1,407 | 337 | 24.0 | **1.18** | 1.07 | 1.30 |  | 604 | 147 | 24.3 | **1.18** | 1.02 | 1.36 |
|  |  | **6<7** | 6,937 | 1,449 | 20.9 | **1.08** | 1.02 | 1.14 |  | 3,537 | 788 | 22.3 | **1.12** | 1.04 | 1.20 |
|  |  | **7<8** | 19,180 | 3,615 | 18.9 | **Ref** |  |  |  | 15,975 | 3,094 | 19.4 | **Ref** |  |  |
|  |  | **8<9** | 12,809 | 2,257 | 17.6 | **0.95** | 0.91 | 1.00 |  | 16,014 | 2,778 | 17.4 | **0.91** | 0.87 | 0.96 |
|  |  | **9<10** | 2,787 | 480 | 17.2 | **0.92** | 0.85 | 1.01 |  | 5,442 | 999 | 18.4 | **0.96** | 0.90 | 1.03 |
|  |  | **10<** | 973 | 199 | 20.5 | **1.01** | 0.89 | 1.14 |  | 1,866 | 440 | 23.6 | **1.16** | 1.06 | 1.27 |
|  | **Bedtime** | |  |  |  |  |  |  |  |  |  |  |  |  |  |
|  |  | **21<24** | 32,644 | 5,430 | 16.6 | **Ref** |  |  |  | 35,493 | 6,128 | 17.3 | **Ref** |  |  |
|  |  | **24<27** | 10,676 | 2,717 | 25.5 | **1.47** | 1.41 | 1.53 |  | 7,315 | 1,998 | 27.3 | **1.51** | 1.45 | 1.58 |
|  |  | **Other** | 773 | 190 | 24.6 | **1.37** | 1.21 | 1.55 |  | 630 | 120 | 19.1 | **1.08** | 0.92 | 1.27 |
|  |  |  |  |  |  |  |  |  |  |  |  |  |  |  |  |
| **Frequency of crying at night (>5 days/week)** | | | | | | |  |  |  |  |  |  |  |  |  |
|  | **Sleep duration** | |  |  |  |  |  |  |  |  |  |  |  |  |  |
|  |  | **<6 hours** | 1,407 | 101 | 7.2 | **1.06** | 0.88 | 1.29 |  | 604 | 41 | 6.8 | **0.97** | 0.72 | 1.31 |
|  |  | **6<7** | 6,937 | 506 | 7.3 | **1.03** | 0.94 | 1.14 |  | 3,537 | 255 | 7.2 | **1.00** | 0.87 | 1.14 |
|  |  | **7<8** | 19,180 | 1,359 | 7.1 | **Ref** |  |  |  | 15,975 | 1,176 | 7.4 | **Ref** |  |  |
|  |  | **8<9** | 12,809 | 948 | 7.4 | **1.05** | 0.97 | 1.14 |  | 16,014 | 1,131 | 7.1 | **0.96** | 0.89 | 1.04 |
|  |  | **9<10** | 2,787 | 198 | 7.1 | **1.02** | 0.89 | 1.18 |  | 5,442 | 397 | 7.3 | **1.00** | 0.90 | 1.12 |
|  |  | **10<** | 973 | 63 | 6.5 | **0.98** | 0.77 | 1.26 |  | 1,866 | 116 | 6.2 | **0.88** | 0.73 | 1.06 |
|  | **Bedtime** | |  |  |  |  |  |  |  |  |  |  |  |  |  |
|  |  | **21<24** | 32,644 | 2,347 | 7.2 | **Ref** |  |  |  | 35,493 | 2,548 | 7.2 | **Ref** |  |  |
|  |  | **24<27** | 10,676 | 771 | 7.2 | **1.03** | 0.95 | 1.12 |  | 7,315 | 516 | 7.1 | **1.00** | 0.91 | 1.10 |
|  |  | **Other** | 773 | 57 | 7.4 | **1.11** | 0.86 | 1.44 |  | 630 | 52 | 8.3 | **1.21** | 0.93 | 1.57 |
| CI, confidence interval; RR, risk ratio | | | |  |  |  |  |  |  |  |  |  |  |  |  |
| ^a^ Adjusted for maternal age at delivery, smoking habits, alcohol consumption, pre-pregnancy body mass index, gestational age at birth, parity, infertility treatment, and infant sex. | | | | | | | | | | | | | | | |

| **Supplemental Table 3**. Comparison of backgrounds between the population analyzed and the population excluded from the analysis due to lack of information about covariates or non-response to any questions about maternal sleep or children’s sleep and development | | | | | | | |
| --- | --- | --- | --- | --- | --- | --- | --- |
|  |  |  |  |  |  |  |  |
|  |  |  | Population analyzed | | Population excluded from the analysis | | p-value (chi-square test) |
|  |  |  | n | % | n* | % |  |
| Total | |  | 73,827 |  | 11,219 |  |  |
| Maternal characteristics | | |  |  |  |  |  |
|  | Age at delivery (years) | |  |  |  |  |  |
|  |  | < 25 | 6,492 | 8.8 | 2,113 | 18.8 | < 0.001 |
|  |  | 25-29 | 20,413 | 27.7 | 3,193 | 28.5 |  |
|  |  | 30-34 | 26,587 | 36.0 | 3,452 | 30.8 |  |
|  |  | ≥ 35 | 20,335 | 27.5 | 2,461 | 21.9 |  |
|  | Smoking habits | |  |  |  |  |  |
|  |  | Never smoked | 44,116 | 59.8 | 4,887 | 47.2 | < 0.001 |
|  |  | Ex-smokers who quit before pregnancy | 17,364 | 23.5 | 2,331 | 22.5 |  |
|  |  | Smokers during early pregnancy | 12,347 | 16.7 | 3,142 | 30.3 |  |
|  | Alcohol consumption | |  |  |  |  |  |
|  |  | Never drank | 25,494 | 34.5 | 3,434 | 32.9 | < 0.001 |
|  |  | Ex-drinkers who quit before pregnancy | 13,326 | 18.1 | 2,338 | 22.4 |  |
|  |  | Drinkers during early pregnancy | 35,007 | 47.4 | 4,669 | 44.7 |  |
|  | Pre-pregnancy body mass index (kg/m^2^) | |  |  |  |  |  |
|  |  | <18.5 | 11,885 | 16.1 | 1,806 | 16.3 | < 0.001 |
|  |  | 18.5–24.9 | 54,635 | 74.0 | 7,800 | 70.2 |  |
|  |  | ≥25.0 | 7,307 | 9.9 | 1,509 | 13.6 |  |
|  | Parity | |  |  |  |  |  |
|  |  | 0 | 32,825 | 44.5 | 4,211 | 38.8 | < 0.001 |
|  |  | ≥ 1 | 41,002 | 55.5 | 6,639 | 61.2 |  |
|  | Infertility treatment | |  |  |  |  |  |
|  |  | No | 68,837 | 93.2 | 10,654 | 95.9 | < 0.001 |
|  |  | Ovulation stimulation/artificial insemination by sperm from husband | 2,750 | 3.7 | 274 | 2.5 |  |
|  |  | Assisted reproductive technology | 2,240 | 3.0 | 184 | 1.7 |  |
|  | Gestational age (weeks) | |  |  |  |  |  |
|  |  | Early term (37-38) | 24,052 | 32.6 | 3,873 | 34.5 | < 0.001 |
|  |  | Full term (39-41) | 49,775 | 67.4 | 7,346 | 65.5 |  |
| Infant sex | | |  |  |  |  |  |
|  |  | Boys | 37,588 | 50.9 | 5,697 | 50.8 | < 0.001 |
|  |  | Girls | 36,239 | 49.1 | 5,515 | 49.2 |  |
| *Numbers in subgroups do not equal overall number because of missing data. | | | | | | |  |
